# Super Stable Ferroelectrics with High Curie Point

**DOI:** 10.1038/srep24139

**Published:** 2016-04-07

**Authors:** Zhipeng Gao, Chengjia Lu, Yuhang Wang, Sinuo Yang, Yuying Yu, Hongliang He

**Affiliations:** 1 National Key Laboratory of Shock Wave and Detonation Physics, Institute of Fluid Physics, China Academy of Engineering Physics, Mianyang 621900, China; 2 Southwest University of Science and Technology, Mianyang 621010, China; 3 Liansu Ltd., 777 Hongqi road, Jiamusi 154002, China

## Abstract

Ferroelectric materials are of great importance in the sensing technology due to the piezoelectric properties. Thermal depoling behavior of ferroelectrics determines the upper temperature limit of their application. So far, there is no piezoelectric material working above 800 °C available. Here, we show Nd_2_Ti_2_O_7_ with a perovskite-like layered structure has good resistance to thermal depoling up to 1400 °C. Its stable behavior is because the material has only 180° ferroelectric domains, complex structure change at Curie point (T_c_) and their sintering temperature is below their T_c_, which avoided the internal stresses produced by the unit cell volume change at T_c_. The phase transition at T_c_ shows a first order behavior which involving the tilting and rotation of the octahedron. The Curie – Weiss temperature is calculated, which might explain why the thermal depoling starts at about 1400 °C.

High-temperature piezoelectric sensing technology is of major importance for the chemical and material processing as well as automotive, aerospace, and power generating industries^1^,^2^. The commercial materials used for high temperature applications such as tourmaline (d_33_ ∼ 1.5 pC/N) can only work until 650 °C^1^,^3^,^4^,^5^. Some ferroelectrics are potential for the high temperature applications due to their high Curie point (T_c_), such as Aurivillius phase ferroelectrics^6^,^7^,^8^, Bi_0.5_Na_0.5_TiO_3_-based ferroelectrics^9^,^10^,^11^, etc. However, all of them could not be used above 700 °C due to their thermal depoling behaviors^6^,^7^. The thermal depoling behaviors of ferroelectrics determine the upper temperature limit of their application as piezoelectrics. Thermal depoling is related to many factors, such as the phase transitions, ferroelectric domain structure, defects and internal mechanical stress^7^,^8^,^12^,^13^,^14^. For lead zirconate titanate ceramics, the internal stress and the non-180° domains reduced the thermal stability of their piezoelectric properties^12^. In the barium titanate system and (1−x)(BiScO_3_)−x(PbTiO_3_) compounds, the internal stress can seriously affect the thermal depoling stability^14^,^15^. Defects in ferroelectrics can interact with domain walls and inhibit their movement at room temperature. However they can be thermally decoupled, which can produce thermal depoling^13^. In Aurivillus phase ferroelectrics, the non-180° domains could reduce the thermal stability^7^,^8^.

The ferroelectrics with perovskite-like layer structure (PLS) ferroelectrics show high Curie point (>1000 °C)^16^,^17^,^18^,^19^. In recent years, many investigations have focused on their potential to be used in high temperature piezoelectric sensor applications^18^,^19^,^20^,^21^. However, there is very limited information on the thermal depoling of PLS phase materials. In the present study, PLS ferroelectric ceramic, Nd_2_Ti_2_O_7_, was investigated and surprisingly it shows a super stable thermal depoling behavior up to 1400 °C. This opens a door for the development of new ferroelectrics for high temperature applications.

## Results and Discussions

**Figure 1:**
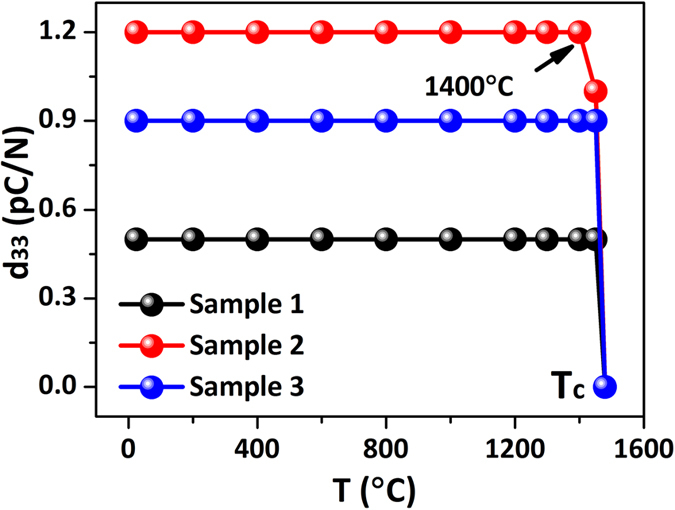
Effect of thermal annealing on piezoelectric properties (d_33_).

**Figure 2:**
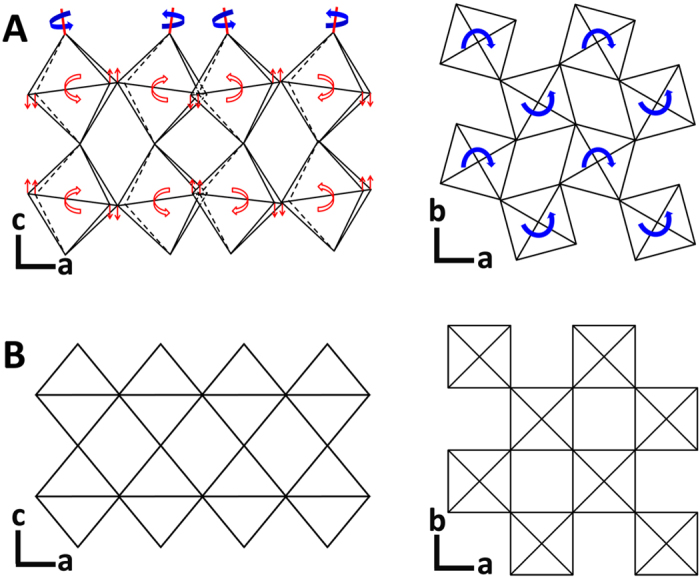
The structure of Nd_2_Ti_2_O_7_ projected along the b- and c-axis for (**A**) ferroelectric phase (P2_1_) and (**B**) paraelectric phase (Cmcm).

Fig. 1A shows the thermal depoling behavior of Nd_2_Ti_2_O_7_ ceramics, in which the piezoelectric constant d_33_, measured at room temperature, are plotted against the annealing temperature. The values of d_33_ of Nd_2_Ti_2_O_7_ samples (1–3) were measured as 0.5, 1.2, and 0.9 pC/N, respectively. The d_33_ value is very stable up to 1400 °C and drop to zero at about 1480 °C, which is the Curie temperature of Nd_2_Ti_2_O_7_. The variation of the d_33_ value is mainly due to the different poling conditions of these three samples. The sample 1, 2 and 3 were poled under the electric field as 20, 27 and 24 kV/mm, respectively. The difference of the poling electrical field is because of the different breakdown field of each sample. During the experiment, the thin ceramic sample was poled in the silicone oil at a temperature of 120 °C under an electric field. We increased the electrical field gradually from 10 kV/mm until the sample was electric breakdown. Therefore, the breakdown field of each sample decides the poling field, which can affect the piezoelectric activity. Compared to other ferroelectric compounds, the thermal depoling temperature of Nd_2_Ti_2_O_7_ is about 600 °C, 800 °C and 1000 °C higher than the values of Aurivillius phases, Bi_0.5_Na_0.5_TiO_3_- based compounds (BNT), and Pb(Zr,Ti)O_3_ compounds (PZT), respectively^6^,^7^,^8^,^13^,^22^,^23^,^24^. This makes Nd_2_Ti_2_O_7_ a great candidate for the high temperature sensor applications, considering its d_33_ is acceptable, which is higher than the d_33_ of commercial piezoelectrics - tourmaline. Actually, this PLS ferroelectric material, Nd_2_Ti_2_O_7,_ exhibits the highest temperature stability of piezoelectric properties among all known ferroelectrics so far as we known. The fact that PLS ferroelectrics only have 180° domains might explain the stability of these compounds. Nd_2_Ti_2_O_7_ has a monoclinic ferroelectric structure with *P2*_*1*_ space group, and the lattice parameters is (a, b, c, β) = (13.020 Å, 5.480 Å, 7.680 Å, 98.3°) as shown in Fig. 2A^21^,^25^,^26^,^27^,^28^,^29^. The paraelectric phase of Nd_2_Ti_2_O_7_, above the T_c_, is orthorhombic lattice with the space group of *Cmcm*^27^,^28^, shown in Fig. 2B. The ferroelectric spontaneous polarization (P_s_) is induced by the rotation of the TiO_6_ octahedron around c-axis (blue arrow) and tilt around b-axis (red arrow), shown in Fig. 2A, which lead the P_s_ only in b-axis, producing 180° domains. In ferroelectric materials, the switching of non-180° domains produces a shape change^30^,^31^. This can lead to large mechanical internal stress in poled materials. These internal stresses combined with thermal activition can produce thermal depoling. These effects are absent in materials with only 180° switching. Additionally, the ferroelectric to paraelectric phase transition of Nd_2_Ti_2_O_7_ involves all of the ions, from the TiO_6_ octahedron rotation and tilt. This characteristic might be another reason for its good stability and high T_c_, due to that the complex structure change increase activation energy of phase transition. Furthermore, the sintering temperature of Nd_2_Ti_2_O_7_ ceramic (1350 °C), which is lower than the T_c_ (1481 °C), can reduce internal stress and increase thermal stability in this ferroelectric ceramic. Because sintering ceramics below its T_c_ can avoid the volume change associated with the Curie transition temperature on cooling from the sintering temperature.

**Figure 3:**
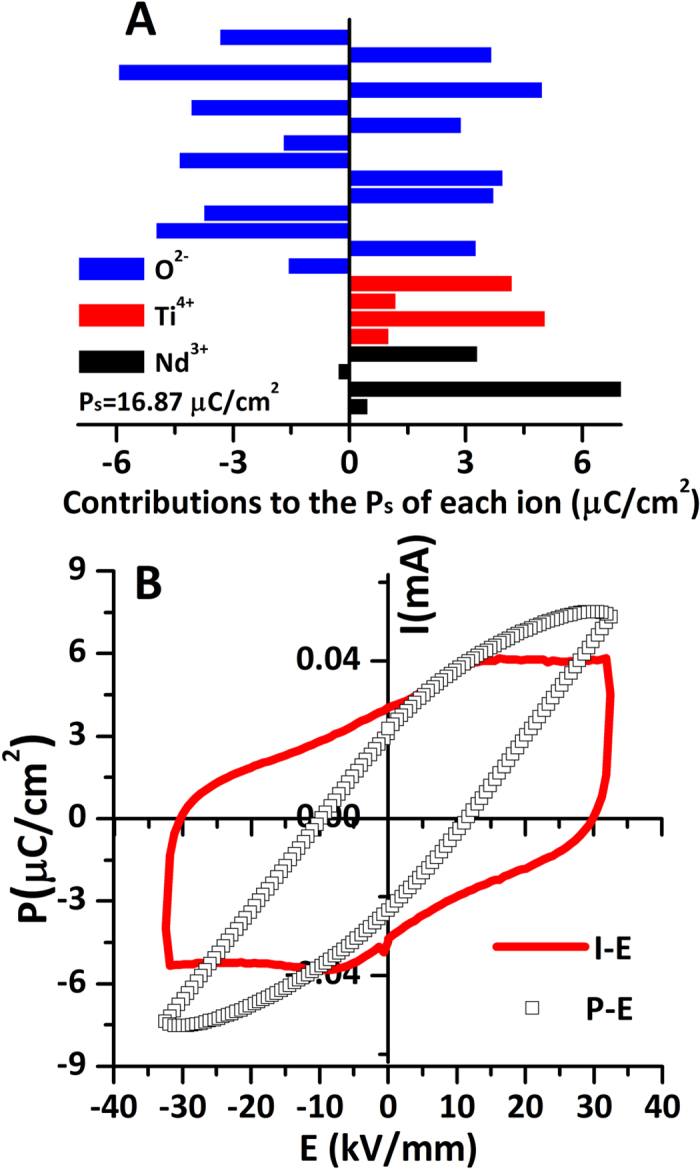
(**A**) Ionic contribution to total spontaneous polarization (P_s_) of each ion of Nd_2_Ti_2_O_7_. (**B**) The polarization – electric field plot (P–E) and current – electric field plot (I–E) measured at a frequency of 5 Hz.

Figure 3A shows the spontaneous polarization (P_s_) value of Nd_2_Ti_2_O_7_ based on the atomic displacements. Ionic displacements along the b-axis from the corresponding positions in the paraelectric structure produce the ferroelectric spontaneous polarization. Displacements along the a- and c- axes are cancelled due to the presence of centro-symmetric centers, which are therefore do not contribute to the total P_s_. Based on the ionic displacements, the total P_s_ of ferroelectric Nd_2_Ti_2_O_7_ was calculated using Shimakawa’s model^8^,^32^:


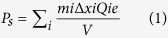


where m_i_ is the site multiplicity and Δx_i_ is the atomic displacement along the c-axis, Q_ie_ is the ionic charge of the ion, and V is the volume of the unit cell. According to the crystal structure parameters of Nd_2_Ti_2_O_7_ reported on the single crystals^23^,^24^,^25^,^26^,^27^,^28^,^29^. the ionic contributions to the total P_s_ are presented in Fig. 3A, and the total P_s_ is calculated as 16.87 μC/cm^2^ at room temperature. Figure 3B shows the polarization – electric field plot (P–E) and current – electric field plot (I–E). From the un-saturated P-E plot, the P_r_ value is about 4.2 μC/cm^2^, which is much smaller than the value calculated. The coercive field is about 10 kV/mm.

**Figure 4:**
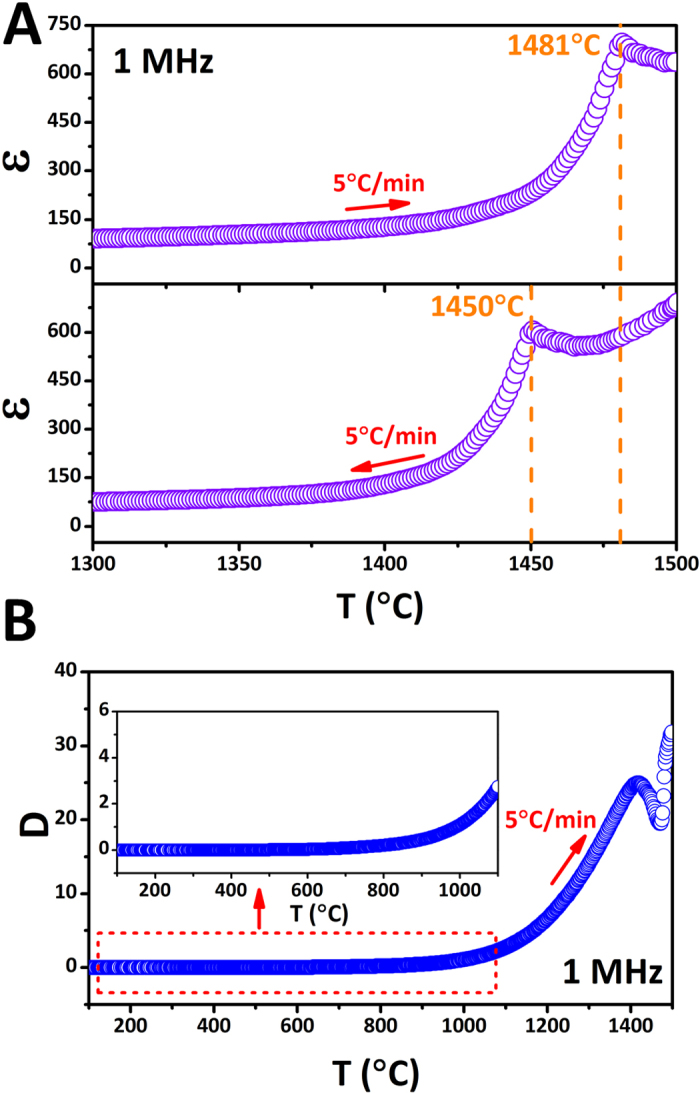
(**A**) Temperature dependence of the dielectric constant of Nd_2_Ti_2_O_7_ at 1 MHz in the processes of heating and cooling. (**B**) The loss (tanθ) measured from 100 °C to 1500 °C at the frequency of 1 MHz, and the insert figure is the enlargement of range from 100 °C to 1100 °C.

**Figure 5:**
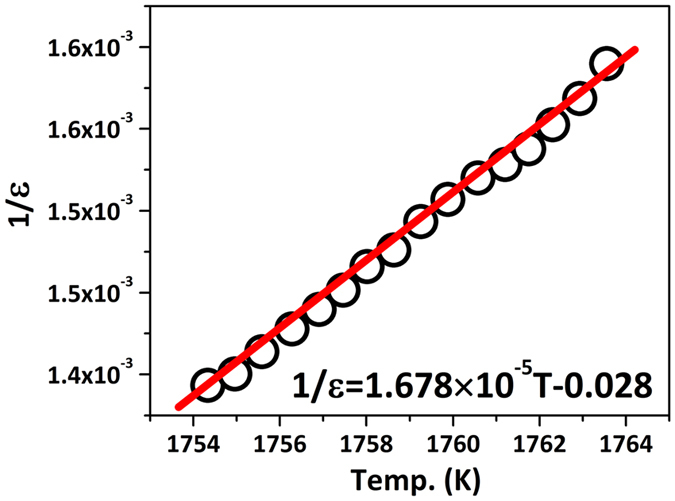
Curie-Weiss fitting for the dielectric constant above the T_c_ for Nd_2_Ti_2_O_7_.

Figure 4A shows dielectric constant (ɛ) of Nd_2_Ti_2_O_7_ ceramic as function of temperature at 1 MHz measured at heating and cooling processes at a rate of 5 °C/min. Typically, the dielectric constant peaks indicate the ferroelectric to paraelectric phase transitions (Curie point, T_c_). The T_c_ at heating process is observed as 1481 °C which is in a good agreement with the literatures^21^,^22^,^33^. However, the T_c_ for the cooling process was different from heating which is 1450 °C. The difference between heating and cooling suggests the ferroelectric to paraelectric transition of Nd_2_Ti_2_O_7_ has thermal hysteresis, which means this is a first order transition. This is also supported by the Curie - Weiss fitting. The function of 1/ε to temperature above T_c_ is shown in Fig. 5 according to the equations 2 and 3, where C is a material-specific Curie constant, T is the absolute temperature, and T_0_ is the Curie - Weiss temperature^31^.


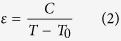



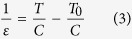


For Nd_2_Ti_2_O_7_, the slope of the fitting is 1.678 × 10^−5^, which is 1/C, and the intercept is the T_0_/C measured as 0.028, and the T_0_ is estimated as 1395 °C, lower than T_c_ in heating process. The T_0_ indicates the temperature point, at which the P_s_ starts going down, and this result might explain why the thermal depoling of Nd_2_Ti_2_O_7_ start at about 1400 °C. The loss (tan θ) at the heating process was shown in Fig. 4B and the insert figure is the enlargement of range from 100 °C to 1100 °C. The loss increase with the temperature increasing, and there is a broad peak just below the T_c_, which can be attributed to ferroelectric domain wall movement. The loss below 1000 °C is small and this is helpful to develop the piezoelectric applications in the future.

In summary, perovskite-like layer structured ferroelectric, Nd_2_Ti_2_O_7_, has super high Curie points and stable piezoelectric properties. The stability of the piezoelectric properties can be explained by their stable ferroelectric domain structure, which consists of only 180° domains; the complex structure at T_c_; and the fact that their sintering temperatures are below T_c_, which avoids the internal strain produced by the volume change at T_c_. The thermal depoling of Nd_2_Ti_2_O_7_ starts at about 1400 °C, which is the Curie - Weiss temperature, at which the P_s_ begin disappearing. This material has the potential to produce a step forward in the maximum operating temperature of ferroelectric/piezoelectric ceramics to >1000 °C.

### Experiment Procedure

**Figure 6:**
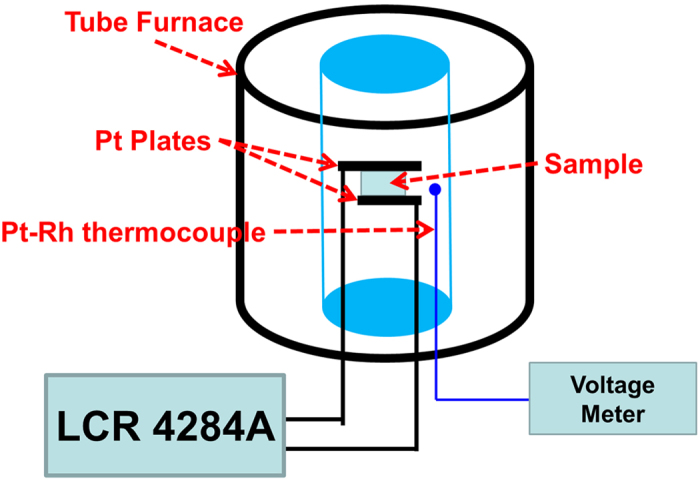
The experimental set up used to measure permittivity and loss at high temperature.

The starting materials were Nd_2_O_3_ (99.9% purity, Alfa Aesar) and TiO_2_ (99.95% purity, Alfa Aesar). The calcination conditions were 1250 °C for 4 h for powder synthesis. The ceramic was fabricated in a spark plasma sintering furnace (HPD 25/1, FCT Systems, Germany) using a two-step method^5^,^6^. The Nd_2_Ti_2_O_7_ powder was sintered at 1150 °C under 80 MPa for 3 min in a 20 mm-diameter graphite die firstly. Then the sintered ceramics were sintered at 1350 °C under 30 MPa for 5 min in a die with 30 mm diameter. X-ray diffraction (XRD, Siemens D5000, Karlsruhe, Germany) patterns was used to detect the phase of the ceramics. Electrodes were fabricated with fired-on platinum paste (Gwent Electronic Materials Ltd, C2011004D5) for electrical properties measurements. The temperature dependence of dielectric constant and loss was measured using a LCR meter (Agilent 4284A) connected to a tube furnace as shown in Fig. 6. The P–E and I–E loops were collected on the ferroelectric test module (TF Analyzer 2000 FE-module, aixACCT, Aachen, Germany). Samples for piezoelectric measurements were poled under various DC electric fields (20–30 kV/mm) in silicone oil at a temperature of 120 °C. We increased the electrical field gradually from 10 kV/mm until the sample was electrical breakdown to obtain high d_33_. Then their piezoelectric constant d_33_ was measured using a quasi-static d_33_ meter (CAS, ZJ- 3B) with the instrument precision of 0.1 pC/N^17^. To confirm the small d_33_ is not an experiment error, both sides of the sample was measured and the d_33_ are positive and negative on each side with the same absolute values. The d_33_ is zero on the un-poled samples as measured. The thermal depoling behavior was investigated by holding the samples at a fixed temperature for 4 hours, then measuring their piezoelectric constant after cooling.

## Additional Information

**How to cite this article**: Gao, Z. *et al*. Super Stable Ferroelectrics with High Curie Point. *Sci. Rep*. **6**, 24139; doi: 10.1038/srep24139 (2016).

## References

[b1] Damjanovic D. Materials for high temperature piezoelectric transducers. Curr. Opin. Solid St. M. 3, 469–473 (1998).

[b2] Turner R. C., Fuierer P. A., Newnham R. E. & Shrout T. R. Materials for high - temperature acoustic and vibration sensors - a review. Appl. Acoust. 41, 299–324 (1994).

[b3] Shekhar Pandey C., Jodlauk S. & Schreuer J. Correlation between dielectric properties and chemical composition of the tourmaline single crystals. Appl. Phys. Lett. 99, 3 (2011).

[b4] Zhang S. J. . High-temperature piezoelectric single crystal ReCa_4_O(BO_3_)_3_ for sensor applications. IEEE Trans. Ultrason. Ferroelectr. Freq. Control. 55, 2703–2708 (2008). 19126494 10.1109/TUFFC.2008.985

[b5] Tressler J. F., Alkoy S. & Newnham R. Piezoelectric sensors and sensor materials. J. Electroceram. 2, 257–272 (1998).

[b6] Yan H. . Effect of texture on dielectric properties and thermal depoling of Bi_4_Ti_3_O_12_ ferroelectric ceramics. J. Appl. Phys. 100, 076103 (2006).

[b7] Yan H. Zhang H., Reece M. & Dong X. Thermal depoling of high curie point aurivillius phase ferroelectric ceramics. Appl. Phys. Lett. 87, 082911 (2005).

[b8] Yan H. . A lead - free high curie point ferroelectric ceramic, CaBi_2_Nb_2_O_9_. Adv. Mater. 17, 5 (2005).

[b9] Kruzina T. V., Sidak V. M., Trubitsyn M. P., Popov S. A. & Suchanicz J. Thermal treatment and dielectric properties of Na_0.5_Bi_0.5_TiO_3_ single crystal. Ferroelectrics 462, 140 (2014).

[b10] Dorcet V., Trolliard G. & Boullay P. Reinvestigation of phase transitions in Na_0.5_Bi_0.5_TiO_3_ by TEM. Part I: First order rhombohedral to orthorhombic phase transition. Chem. Mater. 20, 5061–5073 (2008).

[b11] Trolliard G. & Dorcet V. Reinvestigation of phase transitions in Na_0.5_Bi_0.5_TiO_3_ by TEM. Part II: Second order orthorhombic to tetragonal phase transition. Chem. Mater. 20, 5074–5082 (2008).

[b12] Wan S. & Bowman K. Thermal depoling effects on anisotropy of lead zirconate titanate materials. J. Am. Ceram. Soc. 81, 2717–2720 (1998).

[b13] Zeng T., Yan H. X. & Reece M. J. Effect of point defects on thermal depoling behavior of bismuth layer-structured ferroelectric ceramics. J. Appl. Phys. 108, 9 (2010).

[b14] Hao J., Bai W., Li W. & Zhai J. Correlation between the microstructure and electrical properties in high-performance (Ba_0.85_Ca_0.15_)(Zr_0.1_Ti_0.9_)O_3_ lead-free piezoelectric ceramics. J. Am. Ceram. Soc. 95, 6 (2012).

[b15] Chen S., Dong X. L., Mao C. L. & Cao F. Thermal stability of (1−x)BiScO_3−x_PbTiO_3_ piezoelectric ceramics for high-temperature sensor applications. J. Am. Ceram. Soc. 89, 3270–3272, (2006).

[b16] Nanamats S., Kimura M., Doi K. & Takahash M. Ferroelectric properties of Sr_2_Nb_2_O_7_ single crystal. J. Phys. Soc. Jpn. 30, 300 (1971).

[b17] Nanamats S., Kimura M. Doi K., Matsushita S. & Yamada N. A new ferroelectric: La_2_Ti_2_O_7_. Ferroelectric. 8, 1, 511–513 (1974).

[b18] Ning H. P., Yan H. X. & Reece M. J. Piezoelectric strontium niobate and calcium niobate ceramics with super-high curie points. J. Am. Ceram. Soc. 93, 1409–1413 (2010).

[b19] Gao Z. P., Yan H. X., Ning H. P. & Reece M. J. Ferroelectricity of Pr_2_Ti_2_O_7_ ceramics with super-high curie point. Adv. Appl. Ceram. 112(2), 69–74 (2013).

[b20] Gao Z. P. . Piezoelectric and dielectric properties of Ce substituted La_2_Ti_2_O_7_ ceramics. J. Euro. Ceram. Soc. 33, 1001–1008, (2013).

[b21] Lichtenberg F., Herrnberger A. & Wiedenmann K. Synthesis, structural, magnetic and transport properties of layered perovskite-related titanates, niobates and tantalates of the type A_n_B_n_O_(3n+2)_, A′A_(k−1)_B_k_O_(3k+1)_ and A_m_B_(m−1)_O_3m_. Prog. Solid State. Ch. 36, 253–387 (2008).

[b22] Yan H., Ning H., Kan Y., Wang P. & Reece M. J. Piezoelectric ceramics with super-high curie points. J. Am. Ceram. Soc. 92, 2270–2275 (2009).

[b23] Gomah-Pettry J. R., Sai. S., Marchet P. & Mercurio J. P. Sodium-bismuth titanate based lead-free ferroelectric materials. J. Euro. Ceram. Soc. 24, 1165–1169 (2004).

[b24] Donaji Y., Suarez I. M. & Lee W. E. Relation between tolerance factor and T_c_ in Aurivillius compounds. J. Mater. Res. 16, 3139–3149 (2001).

[b25] Scheunemann K. & Mullerbuschbaum H. Crystal-structure of Nd_2_Ti_2_O_7_. J. Inorg. Nucl. Chem. 37, 2261–2263 (1975).

[b26] Ishizawa N., Marumo F., Kawamura T. & Kimura M. Crystal-structure of Sr_2_Nb_2_O_7_, a compound with perovskite-type slabs. Acta Crystallogr. B 31, 1912–1915 (1975).

[b27] Ishizawa N., Marumo F., Kawamura T. & Kimura M. Compounds with perovskite-type slabs. II. crystal-structure of Sr_2_Ta_2_O_7_. Acta Crystallogr. B 32, 2564–2566, (1976).

[b28] Ishizawa N., Marumo F., Iwai S., Kimura M. & Kawamura T. Compounds with perovskite-type slabs. V. A high-temperature modification of La_2_Ti_2_O_7_. Acta Crystallogr. B 38, 368–372 (1982).

[b29] Ishizawa N., Ninomiya K., Sakakurab T. & Wang J. Redetermination of Nd_2_Ti_2_O_7_: a noncentrosymmetric structure with perovskite-type slabs. Acta Crystall. Sec. E E69, i19 (2013). 10.1107/S1600536813005497 PMC3629463 23633981

[b30] Pöykkö S. & Chadi D. J. Ab initio study of dipolar defects and 180° domain walls in PbTiO_3_. J. Phys. Chem. Solids 61, 4 (2000).

[b31] Jaffe B., Cook W. & Jaffe H. In Non-metallic solids, Piezoelectric Ceramics. Vol. 3 317 (University of Michigan, 1971).

[b32] Shimakawa Y. . Crystal structure and ferroelectric properties of ABi_2_Ta_2_O_9_ (A = Ca, Sr, and Ba). Phys. Rev. B. 61, 6559–6564 (2000).

[b33] Lichtenberg F., Herrnberger A., Wiedenmann K. & Mannhart J. Synthesis of perovskite-related layered A_n_B_n_O_(3n+2)_ = ABO_x_ type niobates and titanates and study of their structural, electric and magnetic properties. Prog. Solid State. Ch. 29, 1–70 (2001).

